# A post-transcriptional mechanism pacing expression of neural genes with precursor cell differentiation status

**DOI:** 10.1038/ncomms8576

**Published:** 2015-07-06

**Authors:** Weijun Dai, Wencheng Li, Mainul Hoque, Zhuyun Li, Bin Tian, Eugene V. Makeyev

**Affiliations:** 1 School of Biological Sciences, Nanyang Technological University, Singapore 637551, Singapore; 2 Department of Microbiology, Biochemistry, and Molecular Genetics, Rutgers New Jersey Medical School, Newark, New Jersey 07103, USA; 3 MRC Centre for Developmental Neurobiology, King's College London, London SE1 1UL, UK

## Abstract

Nervous system (NS) development relies on coherent upregulation of extensive sets of genes in a precise spatiotemporal manner. How such transcriptome-wide effects are orchestrated at the molecular level remains an open question. Here we show that 3′-untranslated regions (3′ UTRs) of multiple neural transcripts contain AU-rich *cis*-elements (AREs) recognized by tristetraprolin (TTP/Zfp36), an RNA-binding protein previously implicated in regulation of mRNA stability. We further demonstrate that the efficiency of ARE-dependent mRNA degradation declines in the neural lineage because of a decrease in the TTP protein expression mediated by the NS-enriched microRNA miR-9. Importantly, TTP downregulation in this context is essential for proper neuronal differentiation. On the other hand, inactivation of TTP in non-neuronal cells leads to dramatic upregulation of multiple NS-specific genes. We conclude that the newly identified miR-9/TTP circuitry limits unscheduled accumulation of neuronal mRNAs in non-neuronal cells and ensures coordinated upregulation of these transcripts in neurons.

## Abstract (web summary)

Nervous system development relies on coherent up-regulation of extensive genes in a precise spatiotemporal manner. Here, the authors show that miR-9/TTP circuitry ensures coordinated up-regulation of neuronal mRNAs in neurons and limits unscheduled accumulation of these transcripts in non-neuronal cells.

## Graphical abstract

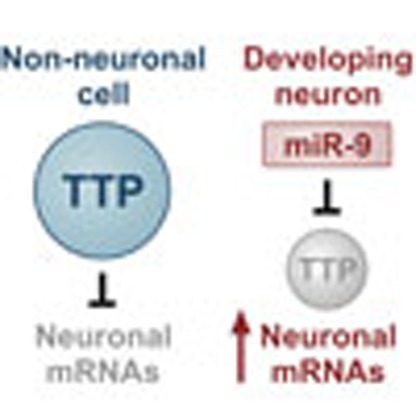

Eukaryotic gene expression is an intricate balancing act between transcription and post-transcriptional steps of RNA metabolism. Developmental readjustment of this balance allows large cohorts of genes to be expressed in cell- and tissue-specific manner. Among other regulators, RNA-binding proteins (RBPs) provide an important means for modulating RNA processing and turnover in the context of cellular differentiation^1^,^2^,^3^,^4^. For example, downregulation of the ubiquitously expressed RBP Ptbp1/PTB/hnRNAP I in the developing brain stimulates neuron-specific alternative pre-mRNA splicing patterns and stabilizes a subset of neuronal transcripts^5^,^6^.

We have previously shown that Ptbp1 levels are dampened in developing the nervous system (NS) by the microRNA miR-124, a non-coding molecule base pairing with partially complementary sites in the Ptbp1 mRNA^7^. Notably, Ptbp1 knockdown is sufficient to induce morphological and functional neuron-like differentiation in non-neuronal cells^8^,^9^. Several important targets have been described for another brain-enriched microRNA, miR-9 (refs 10, 11). However, it is unknown whether the transcriptome-wide effects of miR-9 might be amplified through post-transcriptional mechanisms similar to those implemented in the miR-124/Ptbp1 circuitry.

In contrast to Ptbp1, many RBPs are enriched in the NS^4^,^12^. Of these, the Hu/Elav-like (for example, HuB/Elavl2, HuC/Elavl3 and HuD/Elavl4) and Nova (for example, Nova1 and Nova2) protein families are essential for proper brain development and function^13^,^14^,^15^. NS-specific Hu/Elavl proteins stabilize important neuronal mRNAs including that encoding the axonal Gap43 protein and additionally regulate several neuron-specific pre-mRNA-processing reactions^16^,^17^. Besides their other functions, Nova proteins control a large fraction of neuron-specific alternative splicing events thus diversifying the proteome and modulating steady-state levels of a subset of mRNAs^18^,^19^. Mechanisms ensuring elevated expression of these RBPs in the NS are poorly understood.

A considerable fraction of mammalian transcripts contains 3′ untranslated region (3′ UTR)-localized AU-rich *cis*-elements (AREs)^20^,^21^. These sequences often diminish mRNA stability by recruiting corresponding *trans*-acting RBPs^22^,^23^. Tristetraprolin (TTP/Zfp36), a zinc-finger protein interacting with AUUUA motifs typically within a longer AU-rich context (for example, UAUUUAU), provides an important example of this RBP category^24^. *TTP/Zfp36* knockout (KO) mice develop severe autoimmune/inflammatory phenotypes caused by elevated expression of the tumour necrosis factor (TNF)-α mRNA containing eight UAUUUAU motifs^25^,^26^.

In addition to TNF-α, TTP is known to destabilize other mRNAs encoding a wide range of cytokines, growth factors and proto-oncogenes^24^,^27^. Interestingly, TTP has been implicated in the regulation of HuR/Elavl1, a ubiquitously expressed paralogue of HuB, HuC and HuD^28^,^29^. A recent transcriptome-wide crosslinking and immunoprecipitation survey has suggested that TTP interacts with a substantially larger number of mRNAs^30^. However, what biological processes might rely on this extended repertoire of TTP targets remains unclear.

Here we report that 3′ UTRs of many mRNAs encoding important NS-enriched proteins including neuronal RBPs contain TTP-specific UAUUUAU sequences. We show that activity of the TTP/ARE pathway is diminished in developing NS at least in part because of miR-9-mediated TTP downregulation. This in turn licenses expression of the ARE-containing NS-enriched transcripts. We further show that TTP downregulation is necessary for proper neuronal differentiation *in vitro* and is sufficient for increased expression of UAUUUAU-containing mRNAs in a transformed mouse cell line. Importantly, our analyses of mouse embryonic fibroblasts (MEFs) from wild-type (WT) and TTP KO animals suggest that TTP dampens steady-state levels of an extensive subset of neural mRNAs in non-neural cells *in vivo*. These data implicate TTP as a novel post-transcriptional repressor of NS-specific genes and uncover a molecular mechanism alleviating this repression during brain development.

## Results

### AU-enriched mRNAs tend to accumulate in the neural lineage

We examined previously published microarray data^31^ and detected a significant over-representation of A- an U-rich pentamers in predicted 3′ UTRs^32^ of genes upregulated during embryoid body/retinoic acid (EB/RA)-induced neural differentiation of mouse P19 cells (Supplementary Fig. 1a). Interestingly, the top hits included the three pentamers, UAUUU, UUUAU and AUUUA (Supplementary Fig. 1a and Supplementary Data 1), overlapping with the UAUUUAU motif known to function as a tristetraprolin (TTP/Zfp36)-dependent ARE^24^. Moreover, mRNAs containing one or several ARE cores, AUUUA^21^ were more frequently upregulated in differentiated cultures compared with their AUUUA-less counterparts and this effect was especially pronounced for mRNAs with six or more AUUUA pentamers (Supplementary Fig. 1b,c).

**Figure 1:**
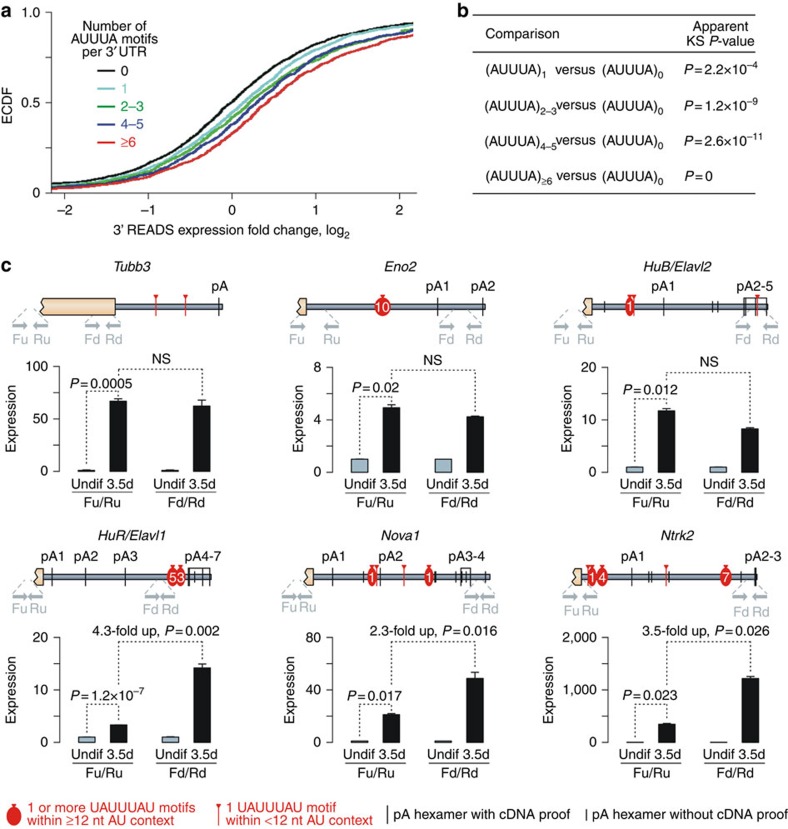
ARE-containing transcripts are frequently upregulated during neural differentiation. (**a**) Empirical cumulative distribution function (ECDF) plots for 3′READS-deduced expression changes in P19 cells undergoing neural differentiation. Individual curves correspond to groups of transcripts with specified numbers of AUUUA motifs within the 3′ UTR. (**b**) Comparison of the transcript groups in **a** using two-sided KS test suggests that mRNAs containing one or several AUUUA motifs are more frequently upregulated than their AUUUA-less counterparts. (**c**) Changes in the expression levels of ARE-containing mRNAs in P19 cells after 3.5 days of EB/RA-induced neural differentiation. Top, 3′ UTR diagrams showing positions of pA sites, AREs and primers used for RT–qPCR analyses. Long black ticks, canonical AAUAAA and AUUAAA pA hexamers occurring within 10–30 nt upstream of the 3′ end in at least one cDNA or EST clone (UCSC Genome Browser) or one-nucleotide modifications of these hexamers used as pA sites in at least five cDNA/EST clones. Short black ticks, AAUAAA and AUUAAA pA hexamers not associated with available cDNA clones. Red ovals, UAUUUAU motifs occurring as a part of ⩾12 nt consecutive AU-nucleotide sequences with the number of individual UAUUUAU heptamers indicated inside the oval. Red ticks, UAUUUAU motifs present within <12 nt AU sequences. Bottom, RT–qPCR relative expression data obtained for undifferentiated (Undif) P19 cultures and cultures differentiated for 3.5 days using upstream (Fu/Ru) or downstream (Fd/Rd) primer pairs. Expression levels in the corresponding undifferentiated samples are set to 1. Data are averaged from three experiments±s.d. and compared by *t*-test.

Neural differentiation involves dramatic changes in mRNA 3′ UTR lengths triggered by globally altered patterns of pre-mRNA cleavage and polyadenylation (APA)^33^,^34^,^35^,^36^. Since it is difficult to distinguish between APA isoforms using microarrays, we analysed transcriptomes of undifferentiated and EB/RA-differentiated P19 cells by the 3′ Region Extraction And Deep Sequencing (3′ READS) procedure recently developed by our group^32^. The newly acquired 3′READS data showed a good correlation with the microarray results (Pearson's correlation coefficient *r*=0.72, *P*<2 × 10^–16^; Supplementary Fig. 1d). Importantly, 3′ UTRs of 3′READS-deduced transcripts upregulated in differentiated P19 cells often contained one or several AUUUA motifs (Fig. 1a,b). Overall, these analyses suggested that ARE-containing transcripts might undergo coordinated upregulation during neural differentiation.

### Many neural mRNAs are tristetraprolin targets

Important examples of mRNAs upregulated in differentiated P19 cells on the basis of the microarray and the 3′ READS data contained two or more UAUUUAU motifs in their 3′ UTRs and encoded neuronal markers (for example, Tubb3, Eno2 and Pcdh19), brain-enriched RBPs (for example, HuB/Elavl2, HuC/Elavl3, HuD/Elavl4 and Nova1) along with neurotrophic receptors and their ligands (for example, Ntrk2 and Bdnf; Fig. 1c and Supplementary Fig. 2). In most of these cases, UAUUUAU motif occurred within a longer stretch of A/U nucleotides (Fig. 1c and Supplementary Fig. 2), a characteristic feature of bona fide AREs^21^,^24^. Our reverse transcription–quantitative PCR (RT–qPCR) analyses with open reading frame-specific primers confirmed that these mRNAs were indeed dramatically upregulated in EB/RA-differentiated P19 cells (Fig. 1c and Supplementary Fig. 2; Fu/Ru and F/R pairs, respectively).

To account for differentiation-induced changes in APA patterns, we re-analysed the above P19 samples using RT–qPCR with primer pairs designed towards downstream 3′ UTR sequences (Fig. 1c; Fd/Rd pairs). For genes with most UAUUUAU sequences preceding a single constitutive cleavage/polyadenylation site (pA; *Tubb3*) or several alternative pA's (*Eno2* and *HuB*), upregulation effects detected using this assay were largely similar to the above Fu/Ru RT–qPCR data (Fig. 1c). However, when UAUUUAU repeats followed proximal alternative pA's, Fd/Rd targets were upregulated to a significantly larger extent than Fu/Ru ones (*HuR/Elavl1*, *Nova1* and *Ntrk2*; Fig. 1c). Of note, the UAUUUAU motifs occurring in the 3′-terminal extension of the long, brain-enriched APA isoform of the HuR mRNA have been previously implicated in TTP-dependent destabilization^28^,^29^,^37^.

**Figure 2:**
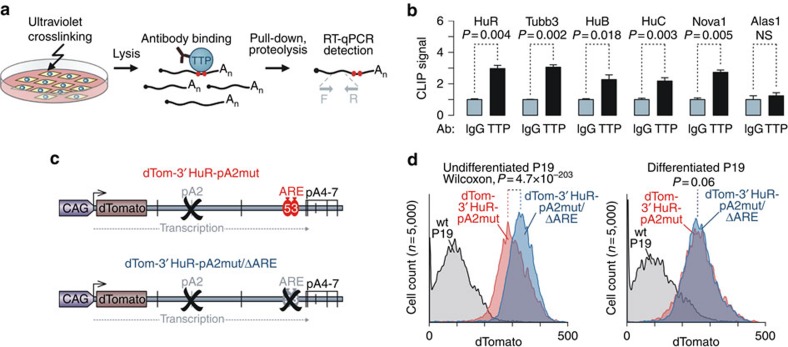
Activity of the TTP/ARE pathway is reduced in cells undergoing neural differentiation. (**a**) CLIP assay diagram (see Methods for details). (**b**) RT–qPCR analysis of mRNA ultraviolet-crosslinked and co-immunoprecipitated with a TTP-specific rabbit antibody from undifferentiated P19 cells. Note that the anti-TTP antibody co-immunoprecipitates significantly larger fractions of ARE-containing mRNA species (HuR, Tubb3, HuB, HuC and Nova1) than corresponding unspecific rabbit IgG control (set to 1). On the other hand, no significant difference between anti-TTP and the control is detected for the Alas1 mRNA encoding aminolevulinic acid synthase 1 and containing no UAUUUAU motifs. Data are averaged from three experiments±s.d. and normalized to background crosslinking signal from Gusb ‘housekeeping' mRNA lacking UAUUUAU's. (**c**) dTomato transgenes fused with PAS2-mutated HuR 3′ UTRs either containing the WT ARE (dTom-3′HuR-pA2mut) or lacking this sequence (dTom-3′HuR-pA2mut/ΔARE). (**d**) Expression levels of the dTomato transgenes introduced in **c** were measured using flow cytometry. Note that expression of dTom-3′HuR-pA2mut/ΔARE is significantly higher than that of dTom-3′HuR-pA2mut in undifferentiated P19 but not in EB/RA-differentiated P19 cells. Background fluorescence of unmodified P19-A9 cells is plotted for reference.

Notably, when we analysed a subset of the above mRNAs (Tubb3, HuB, HuC, HuD and Nova1) expressed in undifferentiated P19 cells using ultraviolet crosslinking/immunoprecipitation (CLIP) with TTP-specific antibodies, TTP–mRNA interaction was detected for Tubb3, HuB, HuC and Nova1 (Fig. 2a,b). We could not obtain conclusive HuD-specific data likely because of the extremely low expression of this mRNA in undifferentiated P19 cells. As expected^28^, the anti-TTP antibody generated a robust CLIP signal for the HuR mRNA control (Fig. 2b). On the other hand, no significant TTP binding was detected for the Alas1 mRNA lacking UAUUUAU sequences (Fig. 2b).

Overall, these data indicated that at least a fraction of ARE-containing and TTP-interacting mRNAs was upregulated during neuronal differentiation. Paradoxically, long alternatively cleaved and polyadenylated isoforms containing a larger number of these motifs appeared to be upregulated to a greater extent than their shorter variants with fewer UAUUUAUs.

### The TTP pathway is inactivated during neural differentiation

A parsimonious explanation for the above results would involve a decrease in the efficiency of TTP/ARE-dependent RNA degradation in differentiated P19 cells. To explore this possibility, we generated two *dTomato* cassettes containing HuR 3′ UTRs with mutated pA2, a major pA utilized in proliferating cells^28^ (Fig. 2c). One of these constructs (dTom-3'HuR-pA2mut) contained the WT ARE repeat previously shown to be targeted by TTP^28^, whereas the other one (dTom-3′HuR-pA2mut/ΔARE) had this element deleted (Fig. 2c). Both plasmid-encoded transcripts were expected to terminate predominantly at the downstream pA4-pA7 sites, thus generating long mRNA products with (dTom-3′HuR-pA2mut) or without AREs (dTom-3′HuR-pA2mut/ΔARE)^28^.

The two constructs were then used to generate corresponding single-copy transgenic P19 cells using a previously described procedure^38^. As expected, when we analysed dTomato expression in these cells using flow cytometry, undifferentiated dTom-3′HuR-pA2mut/ΔARE samples showed noticeably higher dTomato levels than undifferentiated dTom-3′HuR-pA2mut cells (Fig. 2d; *P*=4.7 × 10^–203^, Wilcoxon rank-sum test). However, dTomato expression levels in the two transgenic populations became virtually indistinguishable following EB/RA differentiation (Fig. 2d; *P*=0.060, Wilcoxon rank-sum test). We also failed to detect any difference in dTomato expression between neuron-like fractions of the two differentiated cultures positive for neuronal tubulin βIII (TubβIII) marker, a product of the *Tubb3* gene mentioned above (Supplementary Fig. 3; *P*=0.24, Wilcoxon rank-sum test).

Since the above results suggested that the efficiency of ARE-mediated RNA destabilization could be reduced during neuronal differentiation, we introduced the above dTom-3′HuR-pA2mut and dTom-3′HuR-pA2mut/ΔARE plasmids into primary cortical neurons from E15.5 mouse embryos using a transient magnetofection protocol (Supplementary Fig. 4; see Methods for details). Subsequent flow cytometry analyses showed that both neuronal populations expressed dTomato protein at statistically indistinguishable levels (*P*=0.76, Wilcoxon rank-sum test). On the other hand, magnetically transfected primary MEFs expressed significantly larger amounts of dTomato from dTom-3′HuR-pA2mut/ΔARE than from dTom-3′HuR-pA2mut (Supplementary Fig. 4; *P*=1.8 × 10^–180^, Wilcoxon rank-sum test). We concluded that the UAUUUAU-dependent branch of the RNA decay pathway was largely inactive in neurons.

### TTP levels decrease as a result of miR-9 upregulation

**Figure 3:**
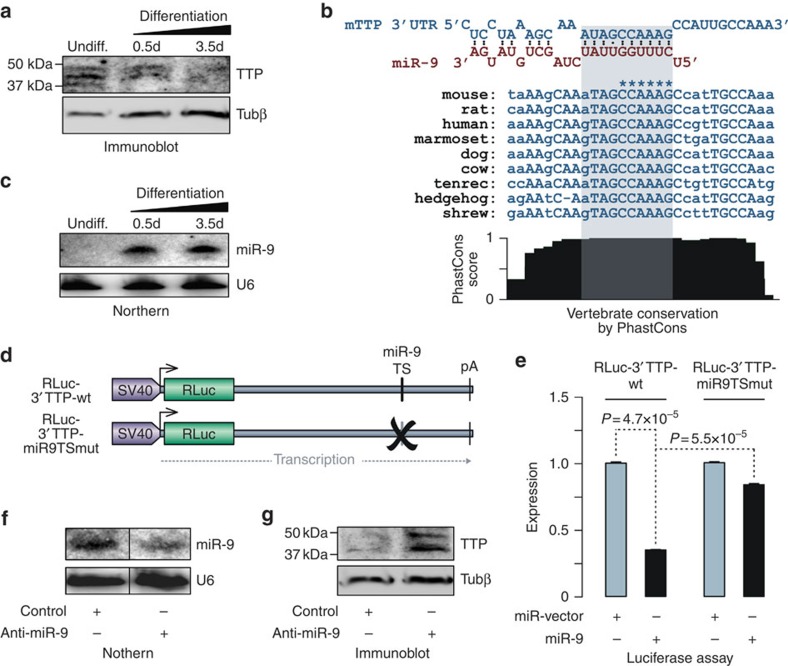
MicroRNA miR-9 reduces TTP expression in neural cells. (**a**) Immunoblot analysis demonstrating a decrease in TTP expression in P19 cells undergoing neural differentiation. Antibody against β-tubulin (Tubβ) is used to control lane loading. (**b**) Top, interaction between miR-9 and the cognate target sequence in the mouse TTP 3′ UTR predicted using RNAhybrid^62^. Middle, interspecies alignment of the target sequences with invariant nucleotides shown in upper case Nucleotides mutagenized to inactivate miR-9-binding are marked by asterisks. Bottom, PhastCons score reflecting probability of sequence conservation across vertebrates^63^. (**c**) Northern blot showing that mature miR-9 levels dramatically increase in P19 cells following EB/RA-induced neural differentiation. U6 RNA is used as a loading control. (**d**) RLuc-3′TTP-wt and RLuc-3′TTP-miR9TSmut Renilla luciferase reporter constructs used in this study. (**e**) HEK293T cells were co-transfected with RLuc-3′TTP-wt or RLuc-3′TTP-miR9TSmut and miR-9 expression plasmid or the corresponding empty vector. Firefly luciferase plasmid pEM231 (ref. 7) was included as a normalization control. Luciferase expression was assayed 24 h post transfection using the Dual-Glo kit (Promega) and the data were processed as recommended. Note that miR-9 dramatically inhibits the RLuc-3′TTP-wt expression while having a scientifically lesser effect on the RLuc-3′TTP-miR9TSmut construct lacking the conserved miR-9 target site. Expression levels of the corresponding miR-vector samples were set to 1. Data are averaged from three experiments±s.d. and compared by *t*-test. (**f**) Following the EB/RA steps, differentiating P19 cells were plated for 12 h and then transfected with a 2′OMe-RNA antisense oligonucleotide against miR-9. Samples were collected 48 h post transfection and the effects of the antisense treatment on the levels of mature miR-9 were analysed with northern blot analysis using U6 RNA as a loading control. (**g**) Immunoblot analysis showing that knockdown of miR-9 carried out as described in **f** leads to noticeable upregulation of the TTP protein.

To test whether reduced efficiency of ARE-dependent mRNA decay in developing neurons could be because of corresponding changes in the TTP expression, we analysed P19 cells undergoing EB/RA differentiation by immunoblotting with a TTP-specific antibody (Fig. 3a). TTP protein levels progressively diminished as a function of differentiation time (Fig. 3a). Immunoblot analysis of mouse embryonic stem cells, neural stem cells and primary cortical neurons further suggested that that protein levels of both TTP and its closely related paralogue BRF1/Zfp36l1 were also dramatically reduced during neurogenesis *in vivo* (Supplementary Fig. 5). Interestingly, TTP appeared to be downregulated at an earlier developmental point than BRF1 (Supplementary Fig. 5). We noticed that the 3′ UTR of TTP–mRNA contained an evolutionarily conserved sequence complimentary to the seed region of microRNA miR-9 (Fig. 3b) known to be expressed in neuronal progenitors and neurons^11^. As expected^39^, mature miR-9 levels increased markedly in differentiating P19 cultures (Fig. 3c), thus suggesting a possible mechanism for TTP downregulation.

To assess whether miR-9 could directly target the predicted site, we fused a Renilla luciferase (RLuc) reporter gene with the TTP 3′ UTR and co-transfected HEK293T cells with this construct (RLuc-3′TTP-wt) and either a miR-9 expression plasmid or the corresponding empty vector (Fig. 3d,e). Satisfyingly, miR-9 reduced RLuc expression ∼2.9-fold (*P*=4.7 × 10^–5^; *t*-test) and mutation of the putative miR-9 target site (RLuc-3′TTP-mir9TSmut) rescued RLuc expression (*P*=5.5 × 10^–5^; *t*-test; Fig. 3e). A similar rescue effect was observed when we treated HEK293T cells co-transfected with RLuc-3′TTP-wt and miR-9 expression plasmid with a target protector oligonucleotide shielding the miR-9 target site in the TTP 3′ UTR (Supplementary Fig. 6a).

To ensure that naturally occurring miR-9 levels were sufficient for the regulation, we transfected differentiated P19 cells with an miR-9-specific antisense 2′-O-meRNA oligonucleotide (Fig. 3f). As expected^40^, this reduced mature miR-9 levels as compared with control-treated cells (Fig. 3f). Notably, miR-9 downregulation resulted in increased TTP protein levels (Fig. 3g). TTP protein levels also noticeably increased in EB/RA-treated P19 cells in response to TTP-specific miR-9 target site protector (Supplementary Fig. 6b). We concluded that TTP expression is reduced during neural differentiation and that this effect is at least in part mediated by miR-9.

### TTP downregulation is required for neuronal differentiation

**Figure 4:**
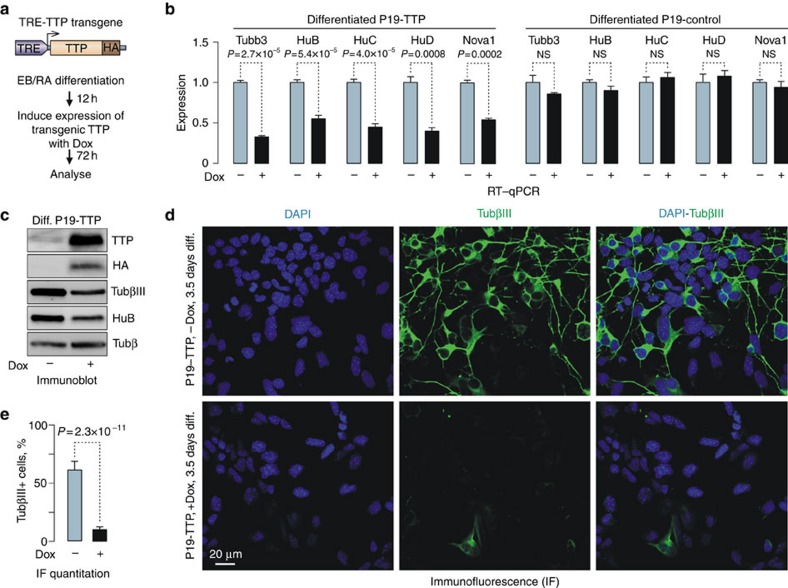
TTP downregulation is essential for neuronal development. (**a**) Dox-inducible TRE-TTP transgene used to express HA-tagged TTP protein in P19 cells. (**b**) RT–qPCR analyses of EB/RA-differentiated P19-TTP cells showing that transgenic TTP protein induced by Dox (Dox+) diminishes expression of TTP target genes compared with the corresponding Dox− samples. Notably, Dox has no effect on TTP targets in control P19 cells. Data are averaged from three amplifications±s.d. and compared by *t*-test. (**c**) Immunoblot analyses of EB/RA-differentiated P19-TTP cells showing that Dox-induced (Dox+) TTP production leads to reduced expression of TubβIII and HuB proteins as compared with the Dox− control. Tubβ is used as a lane loading control. (**d**) Immunofluorescence analyses of P19-TTP cells with and without Dox. (**e**) Quantification of the data in **d** showing that the TTP-expressing (Dox+) culture contains dramatically fewer TubβIII-positive cells than the Dox− control. Data are averaged from 15 randomly selected fields±s.d. and compared by *t*-test.

To determine whether TTP downregulation was required for neuronal differentiation, we prepared P19 cells containing a single-copy HA-tagged TTP transgene driven by a doxycycline (Dox)-inducible promoter and lacking its natural 3′ UTR (TRE-TTP; Fig. 4a). Following the EB/RA induction, TTP expression was induced by Dox and the cells were analysed by RT–qPCR, immunoblotting and immunofluorescence. The RT–qPCR assay suggested that expression of the miR-9-resistant TRE-TTP transgene in differentiated P19 cells led to a significant downregulation of neuronal genes both containing and lacking UAUUUAU repeats in their 3′ UTRs (Fig. 4b and Supplementary Fig. 7a). The immunoblot analysis confirmed Dox-inducible expression of transgenic TTP and showed that this lowered protein levels of two TTP targets, HuB and TubβIII (Fig. 4c), as well as neuronal marker Map2 not predicted to be targeted by TTP (Supplementary Fig. 7b). We also detected a significant decrease in the number of TubβIII-positive cells in EB/RA-differentiated P19 cultures expressing transgenic TTP (Fig. 4d,e).

To address functional significance of reduced TTP expression during neuronal development *in vivo*, we transiently magnetofected primary cortical neurons from E15.5 embryos with a plasmid expressing recombinant TTP from a constitutive promoter (pBS-CMV-TTP; Supplementary Fig. 8a,b). Subsequent RT–qPCR analyses showed that this treatment significantly reduced steady-state levels of UAUUUAU-containing neuronal mRNAs (Tubb3 and HuB) while having no detectable effect on neuronal mRNA lacking UAUUUAU motifs (Map2 and L1cam; S8c-d). Taken together, these results suggested that TTP downregulation in the context of neurogenesis was required for establishing a proper neuronal gene expression programme.

### TTP dampens neuronal mRNAs in neuroblastoma cells

**Figure 5:**
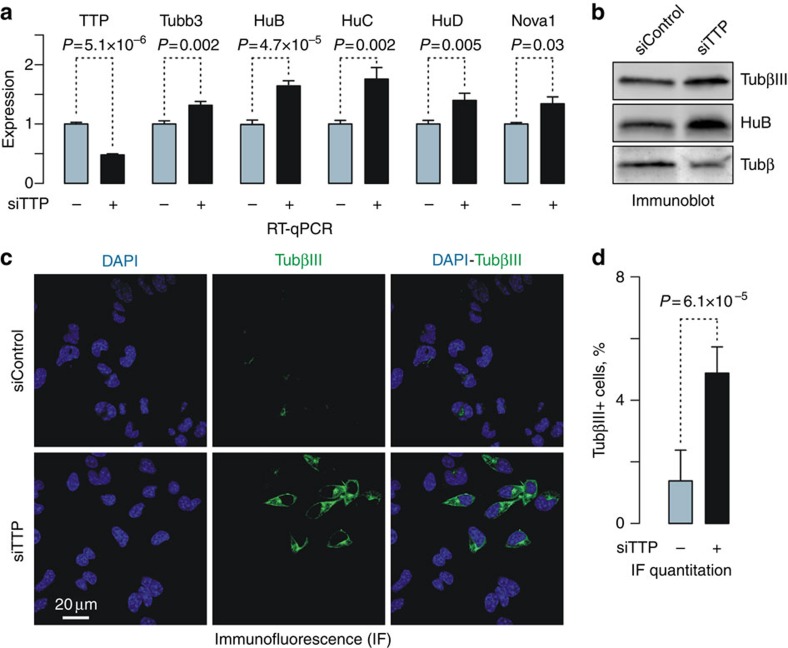
TTP knockdown in neuroblastoma cells is sufficient for upregulation of ARE-containing neuronal markers. (**a**) RT–qPCR analyses showing significant upregulation of TTP targets after knocking down endogenous TTP (siTTP) in Neuro2a cells. Data are averaged from three experiments±s.d. and compared by *t*-test. (**b**) TTP knockdown in Neuro2a cells also stimulates expression of TubβIII and HuB proteins. Tubβ is used as a lane loading control. (**c**) Immunofluorescence analysis demonstrating increased incidence of TubβIII-positive cells in the siTTP-treated sample compared with siControl. (**d**) Quantitation of the data in **c**. Data are averaged from 15 randomly selected fields±s.d. and compared by *t*-test.

To test whether reduced TTP expression was sufficient for upregulation of ARE-containing neuronal mRNAs, we knocked down TTP expression in an easy-to-transfect mouse neuroblastoma cell line, Neuro2a, with a corresponding small interfering (si) RNA (siTTP; Fig. 5a). Compared with cells treated with siControl, treatment with siTTP led to significant upregulation of predicted TTP targets, Tubb3, HuB, HuC, HuD and Nova1, at the mRNA level (Fig. 5a), as well as noticeable accumulation of the HuB and TubβIII proteins (Fig. 5b). Consistent with these data, our IF staining showed that cultures treated with siTTP contained a significantly larger fraction of TubβIII-positive cells than the control-treated ones (Fig. 5c,d). Conversely, overexpression of TRE-TTP in Dox-treated single-copy transgenic Neuro2a cells resulted in a modest but significant decrease in the basal expression levels of Tubb3, HuB, HuC, HuD and Nova1 mRNAs (Supplementary Fig. 9). Thus, TTP limits expression levels of ARE-containing neuronal mRNAs in a transformed non-neuronal cell line.

### *TTP* KO reprogrammes embryonic fibroblast transcriptome

To find out whether TTP could repress neural genes in primary non-neural cells, we turned to the *TTP/Zfp36* KO mouse model^26^. Gene expression patterns of MEFs from the KO (TTP−/−) animals and their WT (TTP+/+) littermates have been previously compared using a microarray approach^41^. The authors treated first-starved-then-serum-stimulated cells expressing TTP at an elevated level with RNA polymerase inhibitor actinomycin D and focused on a subset of transcripts with increased half-lives in the KO cells as compared with the WT. This resulted in identification of 33 TTP targets representing a range of functional categories^41^.

Since the bioinformatics pipeline used in this study could not detect transcripts destabilized by TTP to undetectably low steady-state levels in the WT background but rescued in the KO, we re-analysed the data focusing on genes consistently showing significant expression differences between corresponding KO and WT samples. This yielded 80 genes represented by 100 distinct probes sets that were significantly upregulated and 95 genes represented by 131 probes set that were downregulated across all KO samples (>10-fold effects; Benjamini–Hochberg-corrected *P*<0.05). Strikingly, the upregulated set was significantly enriched for neuron-specific Gene Ontology processes (Supplementary Data 2) and contained Tubb3 and HuB along with several other NS-enriched transcripts (Supplementary Data 3). The upregulated genes tended to be expressed at a level noticeably lower than the transcriptome median in the WT while reaching the median in the KO cells (Supplementary Fig. 10a).

To test whether inactivation of *TTP/Zfp36* might shift the transcriptome of MEF cells towards that of the NS, we utilized the previously described tissue-specific expression ranking approach^42^. Probe sets were first ranked according to their expression levels in the cerebral cortex using an Affymetrix gene expression atlas available for 81 experimentally naive mouse tissues and cell types. The ranks corresponded to position of the cerebral cortex in the list of tissues/cells arranged in an increasing expression order such that genes expressed in cerebral cortex lower than anywhere else were assigned rank 1 and genes expressed higher than anywhere else were ranked 81.

**Figure 6:**
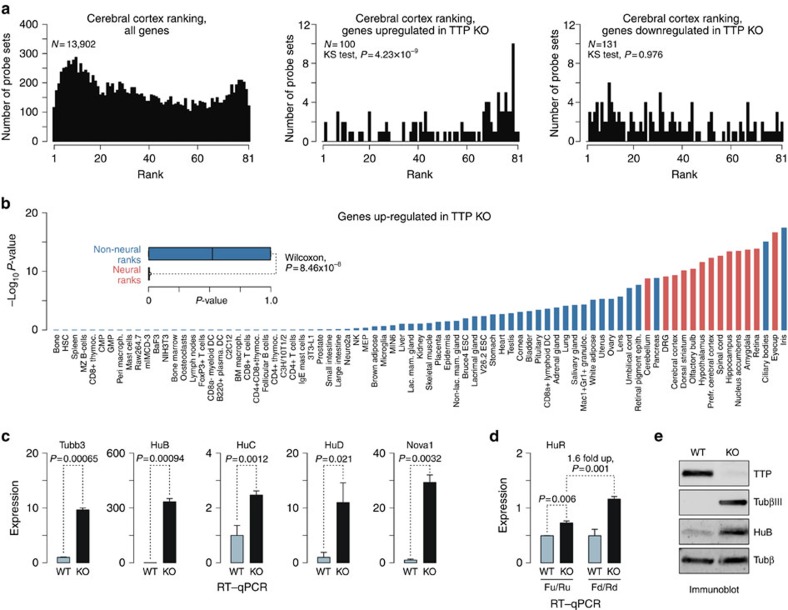
Extensive neural reprogramming of the transcriptome in *TTP*-KO embryonic fibroblasts. (**a**) Distributions of cerebral cortex ranks for all genes expressed in stimulated WT or/and TTP-KO MEFs (left) and for gene groups consistently up- (middle) and downregulated (right) in TTP-KO MEF samples. Note a significant right skew towards high cerebral cortical ranks in the upregulated (*P*=4.23 × 10^−9^; one-sided KS test) but not in the downregulated genes (*P*=0.976; one-sided KS test). (**b**) Distributions of 81 distinct tissue/cell type-specific ranks were plotted for genes upregulated in TTP-KO MEFs and significance of a right skew was estimated using the one-sided KS test introduced in **a**. The graph shows corresponding (−log_10_)-transformed *P* values for each tissue/cell type. Note that the lowest *P* values are observed for neural tissues and non-neural parts of the eye. Overall, Wilcoxon rank-sum test (see inset) shows that neural *P* values are significantly smaller than non-neural ones. Red, neural tissues; blue, non-neural tissues/cells. (**c**) RT–qPCR analysis showing significantly elevated relative expression of TTP targets in untreated TTP KO MEFs compared with similarly prepared WT control. (**d**) RT–qPCR suggesting that the loss of TTP in the KO MEFs leads to accumulation of the long, ARE-containing form of the HuR mRNA. Data in **c,d** are averaged from three experiments±s.d. and compared by *t*-test. WT expression levels are set to 1 (see Fig. 1c for further details). (**e**) Immunoblot analysis showing drastic upregulation of TubβIII and HuB proteins in TTP KO MEFs.

Notably, cerebral cortex rank distribution plotted for upregulated genes was noticeably shifted towards high-ranking genes as compared with the whole array (Fig. 6a and Supplementary Fig. 10b; *P*=4.23 × 10^–9^; one-sided Kolmogorov–Smirnov (KS) test). On the other hand, downregulated genes were statistically indistinguishable from the whole array (Fig. 6a and Supplementary Fig. 10b; *P*=0.976; one-sided KS test). When we repeated this procedure for the rest of the tissues/cell types represented in the atlas, all NS-specific distributions showed significant right shifts for genes upregulated in KO MEFs (Fig. 6b and Supplementary Fig. 10c). NS-specific *P* values were significantly lower than their non-NS counterparts for upregulated (inset in Fig. 6b) but not downregulated genes (Supplementary Fig. 10d). A similar trend was apparent when we analysed changes in the NS and non-NS rank medians (Supplementary Fig. 10e). Taken together, these analyses suggested that TTP repressed a large cohort of neural transcripts in stimulated MEFs.

To examine whether basal TTP expression levels could dampen expression of neural transcripts in non-stimulated fibroblasts, we propagated the WT and KO MEFs kindly provided by the Blackshear laboratory in the presence of 10% of fetal bovine serum (FBS) for several days and analysed the microarray hits using RT–qPCR and immunoblotting. Both Tubb3 and HuB mRNAs were dramatically upregulated in the KO fibroblasts (Fig. 6c) and this effect was also apparent at the protein level (Fig. 6e). Similar upregulation was detected for 10 additional microarray hits with documented NS functions (Supplementary Fig. 11a,b) including Gap43 and Gria3 mRNAs lacking 3′-terminal UAUUUAU motifs but known to be stabilized by Hu/Elavl and Nova proteins, respectively^19^,^43^,^44^.

Although HuC, HuD and Nova1 were not shortlisted with our microarray analysis algorithm, RT–qPCR quantitation showed that these UAUUUAU-containing genes were also significantly upregulated in non-stimulated KO MEFs (Fig. 6c). TTP KO MEFs also expressed increased amounts of the long alternatively cleaved/polyadenylated isoform of the HuR mRNA (Fig. 6d). On the other hand, TTP KO had no effect on the expression levels on the Alas1 ‘housekeeping' mRNA lacking UAUUUAU motifs (Supplementary Fig. 11c). We concluded that one of the TTP functions in non-neural cells *in vivo* could be dampening steady-state expression of multiple neural transcripts.

## Discussion

**Figure 7:**
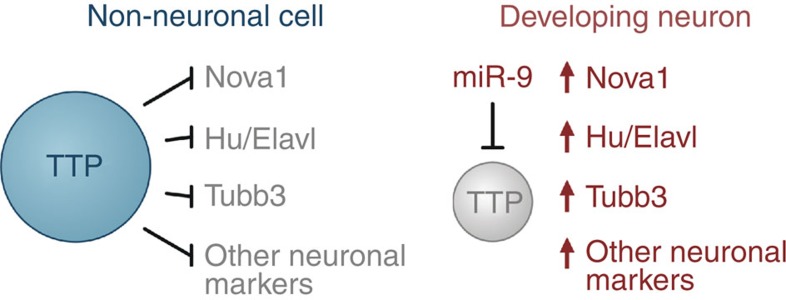
Regulation of NS-specific genes by the miR-9/TTP circuitry.

Our study suggests that, in addition to its well-documented role in destabilizing mRNAs encoding cytokines, growth factors and proto-oncogenes, TTP limits steady-state abundance of a considerable number of ARE-containing neuronal mRNAs in non-neuronal cells (Fig. 7). These transcripts are upregulated in cells undergoing neural differentiation since TTP protein expression is diminished in this context by the brain-enriched microRNA miR-9 (Fig. 7). The experiments with transgenic P19 cells and primary neurons suggest that the newly identified post-transcriptional circuitry is essential for proper neuronal differentiation (Fig. 4 and Supplementary Fig. 8). Further underscoring importance of this mechanism, the newly identified TTP targets include mRNAs encoding RBPs from the Hu/Elavl and Nova families known for their critical contributions to NS development and function as well as mRNAs of essential neuronal markers (for example, TubβIII)^13^,^14^,^15^,^45^.

Data presented in this study (for example, Figs 1c and 6d) further indicate that reduced expression of TTP during neural differentiation may enable expression of 3′-elongated mRNA APA isoforms appearing as a result of frequent skipping of open reading frame-proximal alternative pA sites in the NS^33^,^34^,^35^. Given the major role of the 3′ UTR in defining mRNA translational efficiency, stability and intracellular localization, the impact of the TTP dynamics on the cellular repertoire of APA isoforms warrants further systematic investigation.

The newly identified regulation circuitry is evocative of the previously described miR-124/Ptbp1 switch regulating the choice between non-neuronal and neuronal splicing and mRNA stability patterns^7^. In both cases, a post-transcriptional repressor of neuronal genes is placed under a negative control of a NS-enriched microRNA. This underscores the role of post-transcriptional regulation in differentiating cells and suggests that gene expression changes in this context rely on an extensive crosstalk between microRNA- and RBP-mediated pathways. Interestingly, another well-described example of such double negative regulation logic is provided by the REST/NRSF complex, a transcriptional repressor of neuronal genes in non-neuronal cell that is inactivated in neurons through several molecular mechanisms including the microRNA pathway^10^,^46^,^47^.

Earlier studies have demonstrated that miR-9 and miR-124 might stimulate neurogenesis in a synergistic manner. Indeed, combined expression of these two microRNAs promoted neuronal fate in differentiating mouse ES cell cultures^48^ and triggered detectable *trans*-differentiation of embryonic fibroblasts into neurons^49^. By identifying a global repressor of neuronal mRNA stability as one of the miR-9 targets, our work sheds new light on molecular mechanisms underlying pro-neural activity of this microRNA in mammals.

Although TTP is a critical component of the ARE-dependent mRNA-destabilization machinery, several other RBPs controlling mRNA stability and translation also interact with AREs^22^. Some of these proteins, including TTP paralogues BRF1/Zfp36l1 and BRF2/Zfp36l2, are thought to interact with TTP-specific AREs^24^. Although BRF2 protein was undetectable in cells undergoing neurogenesis, BRF1 was expressed at readily detectable levels early in neurogenesis and dramatically downregulated in neurons (Supplementary Fig. 5). Therefore, it might be interesting—as one of the future directions—to examine possible contribution of BRF1 to post-transcriptional regulation of neural genes.

Yet another line of further studies should focus on the Hu/Elavl protein functions. All four genes encoding mammalian Hu/Elavl paralogues contain 3′-termianal AREs (Fig. 1c and Supplementary Fig. 2) and the steady-state levels of HuB, HuC and HuC, increase markedly on TTP downregulation (Figs 5 and 6). Importantly, Hu proteins are known to interact with U-rich sequences that include but are not limited to AREs recognized by TTP and its paralogues^14^,^30^,^50^. This interaction often stabilizes mRNA targets possibly by minimizing their interaction with repressive RBPs^51^,^52^. Therefore, even partial stabilization of the HuB, HuC and HuD mRNAs triggered by reduced TTP expression may initiate a positive reinforcement mechanism further increasing stability of ARE-containing mRNAs in a Hu/Elavl-dependent manner.

Hu/Elavl protein accumulation would potentially explain the robust upregulation of the Gap43 mRNA in the TTP KO MEFs (Supplementary Fig. 11b) Indeed, this important mRNA is known to be stabilized in neurons through a 3′ UTR U-rich *cis*-element distinct from a TTP-specific ARE but capable of recruiting HuD^43^,^44^. Similarly, initial upregulation of Nova1 protein might account^19^ for the increase in the steady-state level of the Gria3 mRNA lacking discernible AREs (Supplementary Fig. 11b and Supplementary Data 3).

Intriguingly, human Hu/Elavl and Nova have been originally identified as auto-antigens associated with paraneoplastic neurological disorders and members of these protein families are consistently overexpressed in several types of cancers^53^. Moreover, elevated expression of the neural enolase subunit encoded by the *Eno2* gene (Fig. 1c) is commonly used as a neuroendocrine tumour biomarker^54^. Since human orthologues of mouse *HuB*, *HuC*, *HuD*, *Nova1* and *Eno2* genes contain readily discernable AREs and TTP is often downregulated in tumours^27^,^55^,^56^, it will be interesting to examine whether ectopic expression of HuB/C/D, Nova1 and other NS-specific antigens in cancer cells might be triggered by aberrantly low TTP levels. Notably, TTP knockdown was sufficient for HuB/C/D and Nova1 upregulation in Neuro2a neuroblastoma cells (Fig. 5).

In conclusion, our work uncovers a post-transcriptional circuitry dampening expression of multiple neuronal genes in non-neuronal cells and allowing their coordinated upregulation in neurons. This finding may open up new possibilities for improved conversion of non-neuronal cells into neurons for research and therapeutic applications and inform further studies of mechanisms driving overexpression of onconeural antigens by tumour cells.

## Methods

### Plasmids

Mouse TTP-HA expression plasmid (pBS-CMV-TTP) was a gift from Perry Blackshear and pBS-vector control (pBluscriptR) was from RIKEN BioResource Center. New constructs were generated using standard molecular cloning techniques^57^ as outlined in Supplementary Data 4. Site-specific mutations were introduced using modified QuikChange site-directed mutagenesis protocol (Stratagene) using corresponding mutagenic primers (Supplementary Data 5) and KAPA HiFi DNA polymerase (KAPA Biosystems). All primers used in this study are listed in Supplementary Data 5 and plasmid maps and sequences are available on request.

### Cell lines

P19 cells (ATCC) were routinely propagated in P19 growth medium (P19GM) containing α-MEM (Hyclone), 10% FBS (Hyclone, characterized grade), 100 IU ml^−1^ penicillin and 100 μg ml^−1^ streptomycin (Life Technologies). HEK293T and Neuro2a cells (ATCC) were cultured in Dulbecco's modified Eagle's medium (DMEM; Hyclone) containing 10% FBS, 100 IU ml^−1^ penicillin and 100 μg ml^−1^ streptomycin (Life Technologies). The cells were maintained in a humidified incubator at 37 °C and 5% CO_2_.

Mouse TTP was knocked down in cell lines using corresponding ON-TARGETplus mixture containing four proprietary siRNAs designed by Dharmacon/Thermo Scientific. Non-targeting control ON-TARGETplus siRNA was also from Dharmacon/Thermo Scientific. miR-9 was inactivated using either an anti-miR-9 antisense 2′OMe-RNA oligonucleotide (5′- UCAUACAGCUAGAUAACCAAAGA -3′; Dharmacon/Thermo Scientific) or a target protector (Qiagen) against the predicted miR-9-binding sequence within mouse TTP 3′ UTR (5′- CCCUCCUAAAGCAAAUAGCCAAAGCCAUUG -3′). Transfections were carried out using Lipofectamine 2,000 (Life Technologies) as recommended. To transfect cells cultured in a 60-mm dish (4 ml medium), we typically combined 200–500 pmol of an appropriate siRNA or an oligonucleotide with 10 μl of Lipofectamine 2000 pre-diluted with 250 μl of Opti-MEM I reduced serum medium (Life Technologies).

### EB/RA differentiation of P19 cells

To initiate neural differentiation^7^, P19 cells were plated at 1 × 10^5 ^ml^−1^ into bacterial-grade dishes in P19 induction medium (P19IM) containing α-MEM (Hyclone), 5% FBS (Hyclone, characterized grade) and 1 μM of all-*trans*-RA (Sigma). Two days post plating, P19IM was replaced and the EBs were cultured for another 2 days. The EBs harvested from a single 10 cm dish were washed with 1 × PBS and dissociated in 2 ml of 0.25% trypsin–EDTA (Life Technologies) supplemented with 100 μg ml^−1^ DNase I (Roche) for 10 min at 37 °C. The cell suspensions were passed through 100-μm strainers (BD Biosciences) and plated into poly-D-lysine (Sigma)-treated dishes in P19IM lacking RA. Twelve hours after plating, the medium was changed to Neurobasal (Life Technologies) additionally containing 1 × N-2 supplement (Life Technologies) and 2 mM GlutaMAX (Life Technologies) and the cells were allowed to undergo neural differentiation for up to six more days.

### Primary cells

Mouse embryonic neural stem cells were isolated from E14 cortices using NeuroCult Proliferation Kit Mouse (STEMCELL Technologies) and maintained as recommended. Primary neurons were prepared from E15.5 mouse cortices and cultured essentially as in ref. 58, except no astroglial feeders were used for cultures maintained ≤7 days *in vitro* (DIV). Neurons were transfected using NeuroMag reagent (Oz Biosciences) as recommended. Briefly, 2.4 × 10^6^ cortical neurons were plated per 60-mm dish (Corning) pretreated with poly-D-lysine (Sigma). At DIV2–DIV5, 0.1 μg of TTP expression plasmid (pBS-CMV-TTP) or corresponding vector control or 4 μg of a dTomato reporter plasmid (dTom-3′HuR-pA2mut or dTom-3′HuR-pA2mut/ΔARE) was incubated with 6 μl NeuroMag beads in 150 μl Opti-MEM I for 20 min and the mixture was added dropwise to the dish containing neurons. The cultures were then incubated on top of Super Magnetic Plate (Oz Biosciences) for 40 min and incubated for another 40–48 h at 37 °C and 5% CO_2_ before subsequent analyses.

### Flow cytometry

We used natural dTomato fluorescence to sort transgenic P19 cultures and transiently transfected neurons and MEFs. For intracellular staining, cells were trypsinized, washed and fixed at 2 × 10^5^ cells per ml in 1 × PBS containing 2% paraformaldehyde for 15 min at room temperature. Fixed cells were washed twice with 1 × PBS additionally containing 1% BSA. This was followed by incubation with blocking/permeabilization buffer (1 × PBS, 10% horse serum, 1% BSA and 0.15% saponin) for 1 h at room temperature. The cells were then incubated with an anti-Tubb3 (Tuj1) primary antibody diluted in blocking/permeabilization buffer at 4 °C for 1 h. Cells were washed twice with 1 × PBS additionally containing 1% BSA and 0.15% saponin and incubated with a corresponding Alexa-488-conjugated secondary antibody (Life Technologies) diluted 1:1,000 in blocking/permeabilization buffer for 45 min at 4 °C. Cells were finally washed twice with 1 × PBS additionally containing 1% BSA and 0.15% saponin and were analysed using a FACSCalibur flow cytometer (BD).

### Routine molecular biology procedures

Isolation of total RNA, mRNA northern blot analysis, RT–qPCR, immunoblotting, immunofluorescence and luciferase assays were carried out using standard protocols^28^. MicroRNA northern blot analysis^59^ was carried out using an appropriate 5′-[^32^P]-labelled antisense DNA oligonucleotide probes (Supplementary Data 5). RT–qPCR signals obtained using gene-specific primers were normalized to either glyceraldehyde-3-phosphate dehydrogenase or β-glucuronidase housekeeping mRNA controls (Supplementary Data 5; http://www.qiagen.com/spotlight-pages/newsletters-and-magazines/articles/endogenous-controls/). In luciferase reporter assays (Dual-Glo, Promega), *Renilla reniformis* luciferase activity was normalized to that of *Photinus pyralis* firefly luciferase expressed from the pEM231 plasmid^7^. The following antibodies were used for immunoblotting, immunofluorescence, intracellular staining and CLIP: mouse monoclonal anti-β-tubulin (Life Technologies), rabbit polyclonal anti-HA tag (Zymed/Life Technologies), mouse monoclonal anti-Tubb3 (Tuj1; Covance), rabbit polyclonal anti-Map2 (Covance), rabbit polyclonal anti-HuB (Millipore), rabbit polyclonal anti-TTP (a gift from Dr. Blackshear, NIH) and rabbit polyclonal anti-BRF1/BRF2 (Life Technologies). Transgene integration using high-efficiency low-background recombination-mediated cassette exchange^38^. Briefly, P19 acceptor cell line P19-A9 (ref. 38) was co-transfected with a 99:1 mixture of transgene-encoding plasmid and the nlCre expression plasmid (pEM784) and recombinant cells were selected using puromycin.

### RNA-protein CLIP

We investigated TTP–RNA interactions using CLIP protocol modified from ref. 28. Briefly, 10^7^ P19 cells were ultraviolet-crosslinked at 254 nm, 0.4 J cm^−2^ and lysed with the Lysis buffer (50 mM HEPES (pH 7.0), 60 mM KCl, 5 mM MgCl_2_ and 0.5% NP-40, 1 mM dithiothreitol, 0.1 unit per μl rRNasin). The extracts were pre-cleared by incubation with Dynabeads Protein G (Life Technologies) for 2 h at 4 °C and incubated with rabbit anti-TTP antibody or a nonspecific rabbit IgG control at 4 °C overnight. The antigen–antibody complexes were incubated with Dynabeads Protein G for 2 h at 4 °C with constant agitation. The beads were then washed with the Lysis buffer and incubated with the Lysis buffer additionally containing 0.1% SDS and 0.5 mg ml^−1^ proteinase K (Ferments) at 50 °C for 30 min to recover the RNAs from the crosslinked complexes. RNAs were extracted with phenol–chloroform, precipitated with ethanol and assayed using RT–qPCR.

### RNA sequencing

Total RNAs were extracted from undifferentiated and 3.5-day differentiated P19 cells with Trizol (Life Technologies) as recommended and used to prepare 3′READS cDNA libraries^32^. RNA sequencing was carried out using a HiSeq 2500 machine (Illumina). Reads were mapped to the mouse genome (mm9) using Bowtie2 (local mode) and those with mapping quality score ⩾10 were selected for further analyses. Reads with two or more non-genomic A's following the genome-encoded part were qualified as polyadenylation site-supporting (PASS). All PASS reads mapping to the 3′ UTRs were used to calculate corresponding gene expression levels represented as reads per million of total PASS reads. The log_2_-based reads per million ratios between 3.5-day differentiated and undifferentiated samples were used to assess neural differentiation-induced changes in gene expression levels.

### Data analyses

Statistical analyses were performed using Excel and R (http://www.R-project.org/). Images were quantified in ImageJ (http://imagej.nih.gov/ij/). Unless indicated otherwise, data sets were compared using two-tailed *t*-test assuming unequal variance. Published microarray comparison of gene expression in undifferentiated and EB/RA-differentiated neuron-like P19 cells (7 days post EB/RA induction) was downloaded from GEO (accession number GSE23710; (ref. 31)). To analyse motif enrichment in this data set, we used 3′-extended RefSeq gene models assembled as previously described^32^. Enriched motifs were identified using our previously published computer programme PROBE^60^. Differentiation-induced expression changes in gene populations containing specified numbers of AUUUA motifs were compared using two-sided KS test. Flow cytometry data were analysed using the flowCore Bioconductor package (http://www.bioconductor.org/). MicroRNA target sites were predicted using Microcosm Targets resource (http://www.ebi.ac.uk/enright-srv/microcosm/cgi-bin/targets/v5/search.pl). Genes showing consistent expression changes in TTP KO MEFs compared with the corresponding WT littermate controls were identified by re-analysing previously published Affymetrix microarray results ((ref. 41); GEO accession number GSE5324) using gcrma, genefilter and limma Bioconductor packages. Tissue and cell type-specific gene ranks^42^ were computed using the 81 experimentally naive samples from a published gcrma-normalized Affymetrix gene expression atlas ((ref. 61); GEO accession number GSE10246).

## Additional information

**Accession codes:**Newly generated 3′READS data were deposited to the Gene Expression Omnibus (GEO) at the National Center for Biotechnology Information under accession number GSE61098.

**How to cite this article:** Dai, W. *et al*. A post-transcriptional mechanism pacing expression of neural genes with precursor cell differentiation status. *Nat. Commun.* 6:7576 doi: 10.1038/ncomms8576 (2015).

## Supplementary Material

Supplementary Figures and Supplementary References Supplementary Figures 1-11 and Supplementary References

Supplementary Data 1 Top 20 pentamers enriched in 3'UTRs of genes up-regulated upon neural differentiation of P19 cells

Supplementary Data 2 NS-specific genes up-regulated in TTP KO MEFs

Supplementary Data 3 Enrichment for Gene Ontology processes in genes significantly up-regulated in TTP KO MEFs

Supplementary Data 4 Plasmids generated in this study

Supplementary Data 5 Oligonucleotides used in this study
