# Supplementary material for: A post-transcriptional mechanism pacing expression of neural genes with precursor cell differentiation status
Source: Nat Commun. 2015 Jul 6;6:7576. doi: 10.1038/ncomms8576 (PMC4506538; doi:10.1038/ncomms8576)
Supplement: Supplementary Figures and Supplementary References — Supplementary Figures 1-11 and Supplementary References [file ncomms8576-s1.pdf]

a

d

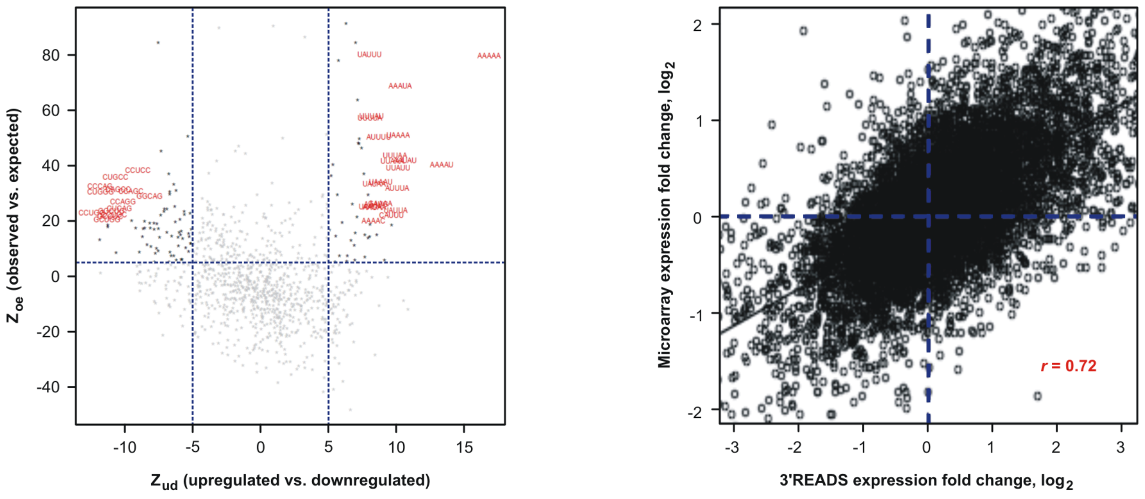

b

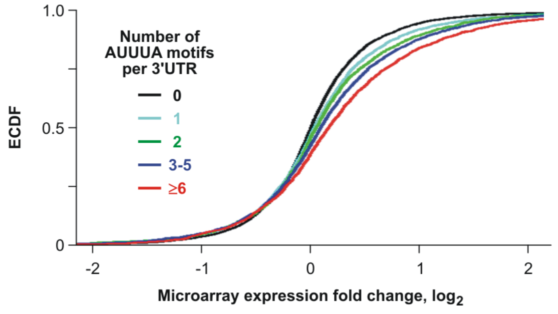

c

Comparison	Apparent KS p-value
$(AUUUA)_1$ vs. $(AUUUA)_0$	$p=5.1\times10^{-6}$
$(AUUUA)_2$ vs. $(AUUUA)_0$	$p=1.1\times10^{-8}$
$(AUUUA)_{3\text{-}5}$ vs. $(AUUUA)_0$	p=0
$(AUUUA)_{\geq6}$ vs. $(AUUUA)_0$	p=0

Supplementary Figure 1: Enrichment of AU-rich motifs in 3'UTRs of genes up-regulated during neural differentiation of P19 cells.

(**a**) Scatter plot showing Z_{oe} and Z_{ud} scores calculated by PROBE [1] for all 3'UTR-specific 5-nucleotide sequences. Z_{ud} (x-axis) is based on a difference in pentamer frequency in up- vs. down-regulated genes from an earlier published microarray study [2] such that a positive Z_{ud} indicates enrichment in up-regulated genes. Z_{oe} (y-axis) reflects a difference between observed vs. expected pentamer frequency. The latter value was calculated by randomizing 3'UTR sequences using a first-order Markov chain model. A positive Z_{oe} value indicates that a pentamer occurs more frequently than expected. Each pentamer is shown as a star and some pentamers with a high Z_{oe} score (including UUUAU, UAUUU and AUUUA) are presented as their actual sequence. (**b**) Empirical cumulative distribution function (ECDF) plots for microarray-deduced gene expression changes in P19 cells undergoing neural differentiation. Individual ECDF curves correspond to gene cohorts with specified numbers of AUUUA motifs within the 3'UTR annotated as described [3]. (**c**) Comparison of the indicated datasets from (b) using two-sided KS test showing significant enrichment of upregulated genes amongst cohorts containing one or several AUUUA motifs vs. their AUUUA-less counterparts. (**d**) Correlation between differentiation-induced gene expression changes detected in P19 cells using microarray and 3'READS analyses.

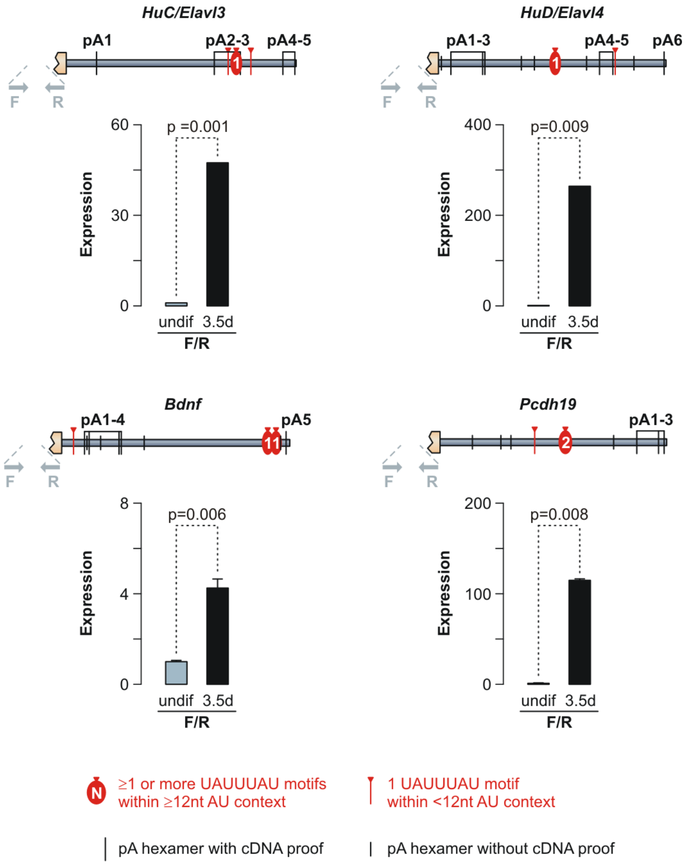

Supplementary Figure 2: Examples of ARE-containing mRNAs significantly upregulated in differentiated P19 cells.

Changes in the expression levels of ARE-containing mRNAs in differentiating P19 cells plated for 3.5 days after the EB/RA induction step. *Top*, 3'UTR diagrams showing positions of pA sites, AREs and primers used for RT-qPCR analyses. Long black ticks, canonical AAUAAA and AUUAAA cleavage/polyadenylation (pA) hexamers occurring within 10-30 nt upstream of the 3' end in least one cDNA or EST clone (UCSC Genome Browser) or one-nucleotide modifications of these hexamers used as pA sites in at least five cDNA/EST clones. Short black ticks, AAUAAA and AUUAAA pA hexamers not supported by available cDNA clones. Red ovals, UAUUUAU motifs occurring as a part of ≥12 nt consecutive AU-nucleotide sequences with the number of individual UAUUUAU heptamers indicated inside the oval. Red ticks, UAUUUAU motifs present within <12 nt AU sequences. *Bottom*, RT-qPCR relative expression data obtained for undifferentiated P19 cultures and cultures differentiated for 3.5 days using corresponding F/R primer pairs. Gene expression levels in the undifferentiated cells are set to 1. Data are averaged from 3 experiments ±SD and compared by t-test.

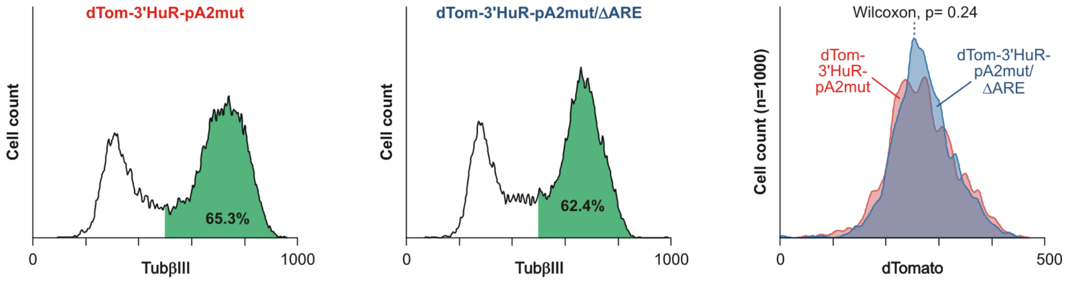

Supplementary Figure 3: ARE-dependent mRNA destabilization is virtually inactive in the TubβIII-positive neuron-like population of differentiated P19 cells.

EB/RA-differentiated transgenic P19 cells introduced in Fig. 2c-d were stained with TubβIII-specific (Tuj1) antibody and analysed by FACS. dTomato expression levels (histogram on the right) were examined in the TubβIII-positive populations selected as shown in the two histograms on the left. No significant difference in dTomato expression was detected between dTom-3’HuR-pA2mut and dTom-3’HuR-pA2mut/ΔARE Tuj1-positive cells (Wilcoxon rank-sum test p=0.24).

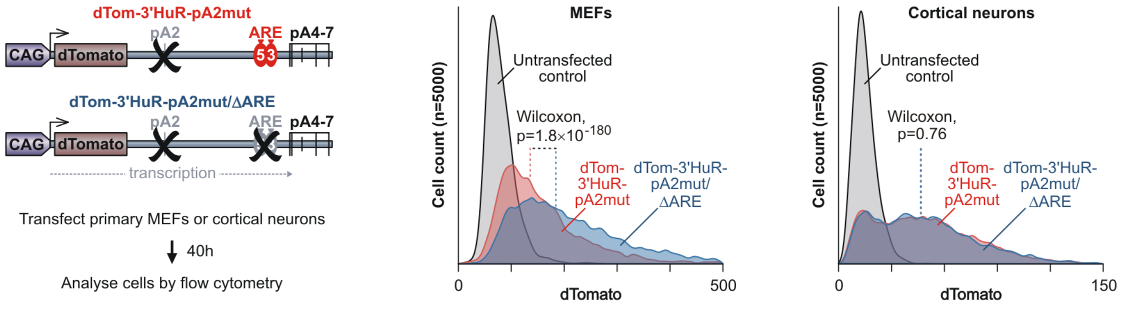

Supplementary Figure 4: ARE-dependent pathway is virtually inactive in primary neurons but readily detectable in embryonic fibroblasts.

Left, Mouse embryonic fibroblasts or primary cortical neurons were magnetically transfected with dTom-3'HuR-pA2mut and dTom-3'HuR-pA2mut/ΔARE constructs and the dTomato protein expression was analysed by flow cytometry. *Right*, no significant expression difference between the two constructs was detected for neurons (Wilcoxon rank-sum test p=0.76), whereas MEFs (*middle*) expressed significantly larger amounts of dTomato from dTom-3'HuR-pA2mut/ΔARE as compared to its ARE-containing counterpart (Wilcoxon rank-sum test $p=1.8\times10^{-180}$).

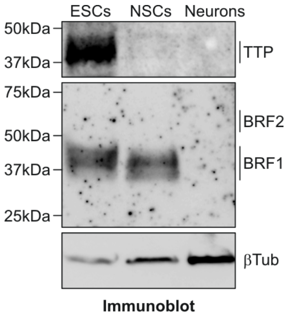

Supplementary Figure 5: Expression levels of TTP protein and its paralog BRF1 decrease during neurogenesis.

Mouse embryonic stem cells (ESCs), neural stem cells (NSCs) and neurons prepared from E15.5 embryonic cortices were analysed by immunoblotting with antibodies recognizing TTP/Zfp36 (*top*) or two of its paralogs, BRF1/Zfp36l1 and BRF2/Zfp36l2 (*middle*). β-tubulin (Tubβ) is used as a lane loading control (*bottom*). Note that TTP and BRF1 are down-regulated during neurogenesis, while BRF2 is not detectable in any of the three cell types.

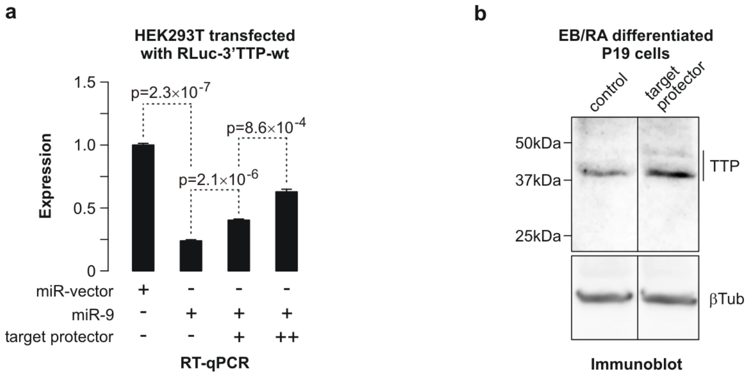

Supplementary Figure 6: miR-9 represses TTP expression by interacting with its 3'UTR-specific target site.

(**a**) HEK293T cells were co-transfected with RLuc-3'TTP-wt and miR-9 expression plasmid or the corresponding empty vector. Firefly luciferase plasmid pEM231 [4] was included as a normalization control. Luciferase expression was assayed 24 hours post transfection using Dual-Glo kit (Promega) and the data were processed as recommended. Two samples were additionally transfected with a TTP 3'UTR miR-9 site-specific target protector (Qiagen) at the final concentration of 50 nM (+) or 100 nM (++). Note that the target protector partially rescues the repressive effect of miR-9. Expression in the RLuc-3'TTP-wt / miR-vector co-transfected sample is set to 1. Data are averaged from 3 experiments ±SD and compared by t-test. (**b**) EB/RA-treated P19 cells were transfected with 125 nM of TTP 3'UTR miR-9 site-specific target protector and the expression of TTP protein was analysed by immunoblotting 48 hours post transfection. β-tubulin (Tubβ) is used as a lane loading control.

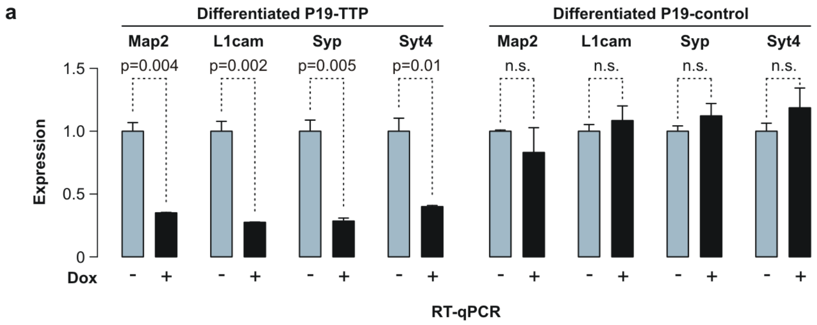

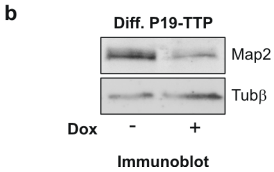

Supplementary Figure 7: TTP down-regulation is necessary for proper neuronal differentiation of EB/RA-induced P19 cells.

(**a**) RT-qPCR analyses of EB/RA-differentiated P19-TTP cells described in Fig. 4. Note that transgenic TTP protein induced by Dox diminishes expression of neuronal marker genes either lacking (Map2 and L1cam) or containing a single UAUUUAU motif in their 3'UTRs (Syp and Syt4), while having no significant effect on these genes in control P19 cells. Data are averaged from 3 amplifications ±SD and compared by t-test. (**b**) Immunoblot analysis of EB/RA-differentiated P19-TTP cells showing reduced expression of Map2 protein in Dox-induced P19-TTP cells as compared to the corresponding Dox(-) control. Antibody against β-tubulin (Tubβ) is used to control lane loading.

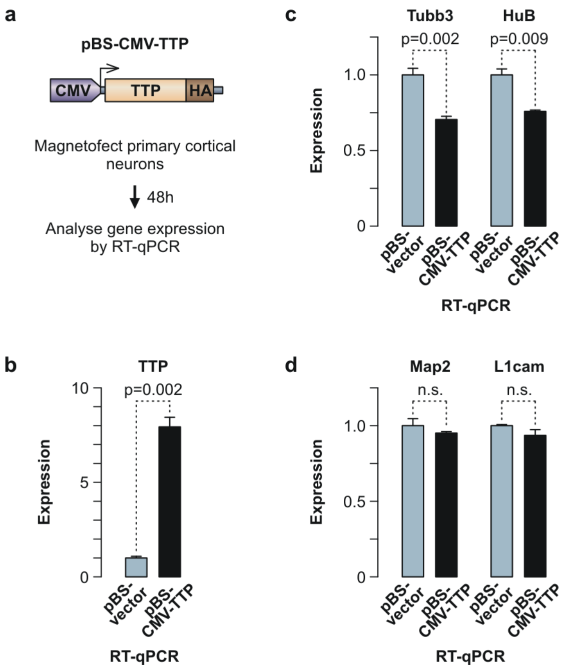

Supplementary Figure 8: TTP down-regulation is necessary for establishing a proper gene expression program in primary cortical neurons.

(**a**) Primary cortical neurons were transiently magnetofected with TTP expression construct (pBS-CMV-TTP) or a pBS-vector control and analysed by RT-qPCR. (**b**) RT-qPCRs analysis showing that pBS-CMV-TTP-transfected neurons express TTP at significantly higher level than the vector control, as expected. (**c-d**) RT-qPCRs showing that transient expression of recombinant TTP (c) significantly reduces levels of Tubb3 and HuB mRNAs containing UAUUUA motifs in their 3'UTRs but (d) has no detectable effect on mRNAs encoding neuronal markers Map2 and L1cam but lacking UAUUUAU motifs. In (b-d), gene expression levels in the vector-transfected samples are set to 1. Data are averaged from 3 experiments ±SD and compared by t-test.

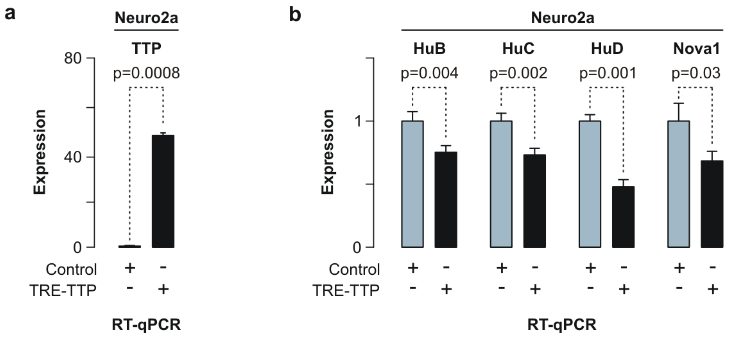

Supplementary Figure 9: TTP over-expression dampens basal expression levels of ARE-containing neuronal mRNAs in neuroblastoma cells.

RT-qPCR analyses of Dox-induced Neuro2a cells either containing or lacking a single-copy of TRE-TTP transgene depicted in Fig. 4a. Note that cells containing the TRE-TTP transgene express (**a**) TTP mRNA at a significantly elevated level and (**b**) mRNAs of the TTP targets at a significantly reduced level as compared to the control. Data are averaged from 3 amplifications ±SD and comared by t-test.

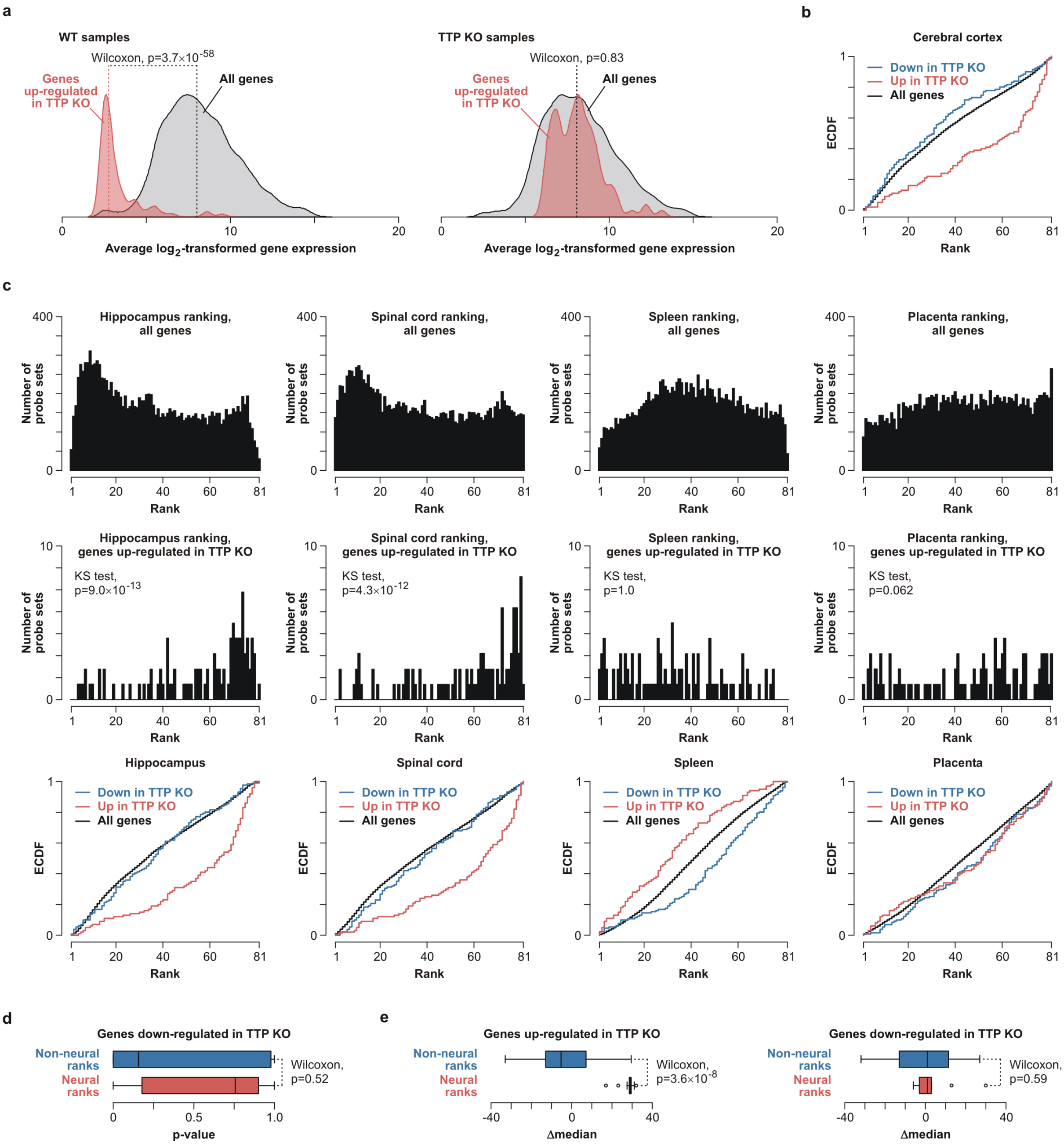

Supplementary Figure 10: Global NS-like reprogramming of the TTP KO MEF transcriptome.

(**a**) Distributions of gene expression levels for all genes (grey) and genes significantly up-regulated in TTP KO MEFs (red). Note that in WT MEFs the up-regulated genes are expressed at significantly lower levels than all genes but in the KO MEFs their expression levels are statistically indistinguishable from all genes. (**b**) ECDF curves for all, up- and down-regulated genes calculated for the cerebral cortex rank distributions in Fig. 6a. (**c**) Distributions of tissue-specific ranks for all genes expressed in stimulated WT or/and TTP-KO MEFs (*top*) and gene groups consistently up-regulated in stimulated TTP-KO MEF samples (*middle*). Corresponding ECDF curves for all, up- and down-regulated genes are shown at the *bottom*. Note significant right shifts in the up-regulated gene ranks for neural (hippocampus and spinal cord) but not for non-neural tissues (spleen and placenta). (**d**) Wilcoxon rank-sum test showing no significant difference between NS and non-NS p-values

calculated for genes down-regulated in TTP KO MEFs. (**e**) Wilcoxon rank-sum comparison of NS and non-NS differences between rank distribution medians for genes with changed expression and all genes (Δmedian). Note that Δmedian values calculated for NS ranks are always positive and that they significantly exceed Δmedian values for non-NS ranks in the case of up-regulated (*left*) but not down-regulated genes (*right*).

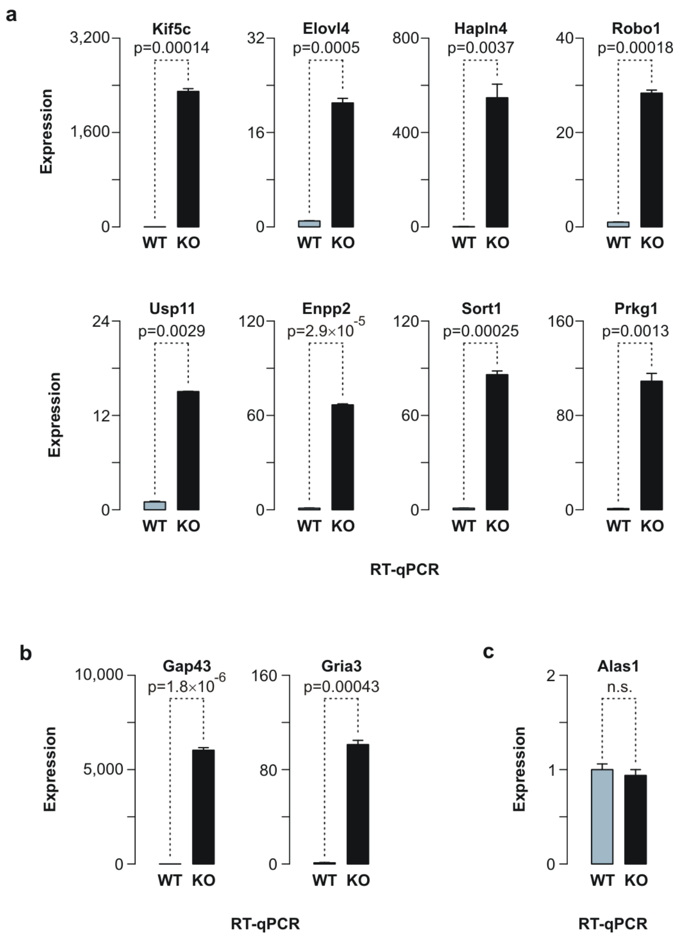

Supplementary Figure 11: Dramatic up-regulation of neural markers in TTP KO MEFs.

RT-qPCR analyses showing significantly elevated expression of NS-enriched genes (**a**) with or (**b**) without 3'-terminal UAUUUAU motifs in untreated TTP KO MEFs as compared to similarly prepared WT control MEFs. (**c**) RT-qPCR detecting no significant effect of TTP KO on the expression levels of the "housekeeping" gene *Alas1* (http://www.qiagen.com/spotlight-pages/newsletters-and-magazines/articles/endogenous-controls/). All data are averaged from 3 amplifications ±SD and compared by t-test.

Supplementary References

1 Hu, J., Lutz, C. S., Wilusz, J. & Tian, B. Bioinformatic identification of candidate cis-regulatory elements involved in human mRNA polyadenylation. *RNA* **11**, 1485-1493, doi:10.1261/rna.2107305 (2005).

2 Suzuki, H. *et al.* Comprehensive analysis of alternative splicing and functionality in neuronal differentiation of P19 cells. *PloS one* **6**, e16880, doi:10.1371/journal.pone.0016880 (2011).

3 Hoque, M. *et al.* Analysis of alternative cleavage and polyadenylation by 3' region extraction and deep sequencing. *Nat Methods*, doi:nmeth.2288 [pii] 10.1038/nmeth.2288 (2012).

4 Makeyev, E. V., Zhang, J., Carrasco, M. A. & Maniatis, T. The MicroRNA miR-124 promotes neuronal differentiation by triggering brain-specific alternative pre-mRNA splicing. *Mol Cell* **27**, 435-448, doi:S1097-2765(07)00488-1 [pii] 10.1016/j.molcel.2007.07.015 (2007).
